# Ensembl 2018

**DOI:** 10.1093/nar/gkx1098

**Published:** 2017-11-16

**Authors:** Daniel R Zerbino, Premanand Achuthan, Wasiu Akanni, M Ridwan Amode, Daniel Barrell, Jyothish Bhai, Konstantinos Billis, Carla Cummins, Astrid Gall, Carlos García Girón, Laurent Gil, Leo Gordon, Leanne Haggerty, Erin Haskell, Thibaut Hourlier, Osagie G Izuogu, Sophie H Janacek, Thomas Juettemann, Jimmy Kiang To, Matthew R Laird, Ilias Lavidas, Zhicheng Liu, Jane E Loveland, Thomas Maurel, William McLaren, Benjamin Moore, Jonathan Mudge, Daniel N Murphy, Victoria Newman, Michael Nuhn, Denye Ogeh, Chuang Kee Ong, Anne Parker, Mateus Patricio, Harpreet Singh Riat, Helen Schuilenburg, Dan Sheppard, Helen Sparrow, Kieron Taylor, Anja Thormann, Alessandro Vullo, Brandon Walts, Amonida Zadissa, Adam Frankish, Sarah E Hunt, Myrto Kostadima, Nicholas Langridge, Fergal J Martin, Matthieu Muffato, Emily Perry, Magali Ruffier, Dan M Staines, Stephen J Trevanion, Bronwen L Aken, Fiona Cunningham, Andrew Yates, Paul Flicek

**Affiliations:** European Molecular Biology Laboratory, European Bioinformatics Institute, Wellcome Genome Campus, Hinxton, Cambridge CB10 1SD, UK; Eagle Genomics Ltd., Wellcome Genome Campus, Hinxton, Cambridge CB10 1DR, UK; Wellcome Trust Sanger Institute, Wellcome Genome Campus, Hinxton, Cambridge CB10 1SA, UK

## Abstract

The Ensembl project has been aggregating, processing, integrating and redistributing genomic datasets since the initial releases of the draft human genome, with the aim of accelerating genomics research through rapid open distribution of public data. Large amounts of raw data are thus transformed into knowledge, which is made available via a multitude of channels, in particular our browser (http://www.ensembl.org). Over time, we have expanded in multiple directions. First, our resources describe multiple fields of genomics, in particular gene annotation, comparative genomics, genetics and epigenomics. Second, we cover a growing number of genome assemblies; Ensembl Release 90 contains exactly 100. Third, our databases feed simultaneously into an array of services designed around different use cases, ranging from quick browsing to genome-wide bioinformatic analysis. We present here the latest developments of the Ensembl project, with a focus on managing an increasing number of assemblies, supporting efforts in genome interpretation and improving our browser.

## INTRODUCTION

Ensembl's purpose is to accelerate genomic research worldwide and amplify the impact of new discoveries by providing an openly-accessible window into the wealth of data produced by the scientific community. Genomic and epigenomic datasets are selected from public archives such as INSDC (1), ENA (2), dbSNP (3) or EVA (https://www.ebi.ac.uk/eva), downloaded, then processed by our multiple automated analysis methods. Their results are finally stored into an integrated array of databases and tailored storage solutions that are read by various APIs and utilities. Our web-based genome browser (http://www.ensembl.org) is in effect the visible tip of a very large underlying infrastructure.

The Ensembl project has grown with the field of genomics since the first releases of the draft human genome (4), when we launched our initial visualisation of the genomic sequence and the location of the genes within it (5). As the field expanded, so did Ensembl. Starting with mouse as the second sequenced vertebrate genome, we developed comparative genomic resources that now encompass multiple whole genome alignments and gene-level phylogenetic trees (6). Data from large variation discovery projects such as HapMap (7) and the 1000 Genome Project (8) were incorporated into our variation storage and annotation resources (9). International epigenomic surveys, including ENCODE (10) and Blueprint (11), provided supporting evidence for our genome-wide annotation of regulatory elements (12). This expansion has led us to collaborate directly with major bioinformatics databases and resources such as UniProt (13), GENCODE (14), UCSC and NCBI (15).

In parallel, Ensembl is used in increasingly sophisticated ways. Our infrastructure now underlies an array of services for many different use cases. For quick queries, our web browser is likely the tool of choice. For genome-wide analyses, a few lines of code are sufficient to connect any computer to our databases via the Ensembl application programming interfaces (APIs, 16,17). Finally, for common data analysis workflows, we support dedicated tools, such as BioMart (18) and the Ensembl Variant Effect Predictor (VEP, (19)).

Our work has always been led by the firm conviction that scientific progress can only be accelerated by making data freely available as early as possible. This philosophy led to us adopting the FAIR principles of Findable, Accessible, Interoperable and Reusable (20) long before these were formalised. We define globally unique and persistent identifiers for our genes and other genomic features in our databases, our data are freely and programmatically available, we adhere to international naming standards and ontologies, and we track and credit the provenance of all our annotations. In addition to serving the community, these practices have the pragmatic effect of supporting our aim to develop a sustainable ecosystem of data services that can be automatically combined. Keeping up with the fast pace of genomics has required that our resources be integrated and inter-compatible.

Now that next-generation sequencing (NGS) is commonplace in many laboratories and that efficient bioinformatics toolkits have been developed, knowledge extraction is the bottleneck of genomics (21). Genomes are no longer sequenced one by one, rather in batches, and already Ensembl can display clades, as illustrated by our addition in 2016 of a collection of laboratory mouse strains (22). Furthermore, NGS machines are making their way into clinical laboratories and personal genotyping is becoming routine. To better support genome interpretation, we regularly enrich our annotation of variants. Finally, we are constantly improving our usability, for example by regularly refreshing our web interface with interactive selection tools to help guide visitors through the many available options and datasets.

## COVERING MORE GENOMES

Ensembl release 90 (August 2017) included 15 new and updated annotated rodent genomes including two assemblies of the Chinese hamster ovary (CHO) cell line, male and female genome assemblies for naked mole-rat (*Heterocephalus glaber*), and three chromosome-level assemblies (*Mus pahari, Mus caroli* and *Microtus ochrogaster*). We generated these annotations using a combination of annotation mapping (via whole genome alignment) from the *Mus musculus* GENCODE gene set (14), alignments of a targeted subset of UniProt (13) vertebrate protein sequences and, where available, RNA-seq data. For *Mus caroli* and *Mus pahari* we imported the annotations generated by the Mouse Genomes Project (23). These new and updated rodent genomes join the 16 mouse strains whose annotations were imported in Ensembl release 86 (October 2016) (22).

We also annotated the newest pig reference assembly (Sus scrofa 11.1) using a combination of Illumina data from 28 tissues and PacBio IsoSeq data from nine tissues. With this wealth of transcriptome data, we annotated over 25 000 genes with almost 50 000 transcript isoforms, a significant increase when compared to the previous assembly. The annotation generated from individual tissues for both the Illumina and PacBio data are viewable as tracks in the browser and accessible via the API. We plan to update the gene count in subsequent releases by including manual annotation mapped from the previous assembly and more long non-coding RNA (lncRNA) annotation.

We updated the annotation of the GRCz10 zebrafish assembly using the extensive set of RNA-seq data aligned to the genome since the original annotation was completed, including the transcriptomes of 18 different developmental stages. These data added new UTRs, transcript isoforms and genes, including over 2000 lncRNAs. In addition, this final update to the GRCz10 gene set included a track of primary miRNA transcripts, assembled de novo using RNA-seq data and then mapped to the genome (https://www.biorxiv.org/content/early/2017/02/20/107631). These transcripts are viewable in the browser and can be compared to the existing zebrafish annotation across the genome.

We regularly updated the GENCODE gene set (14) for both mouse and human over the past year. Mouse, which is currently the subject of GENCODE’s intensive manual annotation effort, has been updated every release. Because a first pass of manual annotation has been completed for human, updates are currently applied every other release only.

We annotated two different assemblies of the Chinese hamster ovary cell line CHOK1: CriGri_1.0 (GCA_000223135.1) and CHOK1GS_HDv1 (GCA_900186095.1). Both annotations were produced with the same pipeline but different inputs. First, the CriGri_1.0 assembly is older (released in 2011) than CHOK1GS_HDv1 (2017). Second, on CriGri_1.0 we used a selection of transcriptomic data from the European Nucleotide Archive, whereas for CHOK1GS_HDv1 we used transcriptomic data specifically produced for this annotation by the assembly provider and available at http://www.ebi.ac.uk/ena/data/view/PRJEB14303. Despite these differences in inputs, the two annotations contained similar numbers of genes in each category (except for lncRNA genes which will be added to CHOK1GS_HDv1 in a future release of Ensembl). Nonetheless, the CHOK1GS_HDv1 assembly had slightly more genes overall, likely reflecting the use of more recent genomic and transcriptomic data.

**Figure 1.**
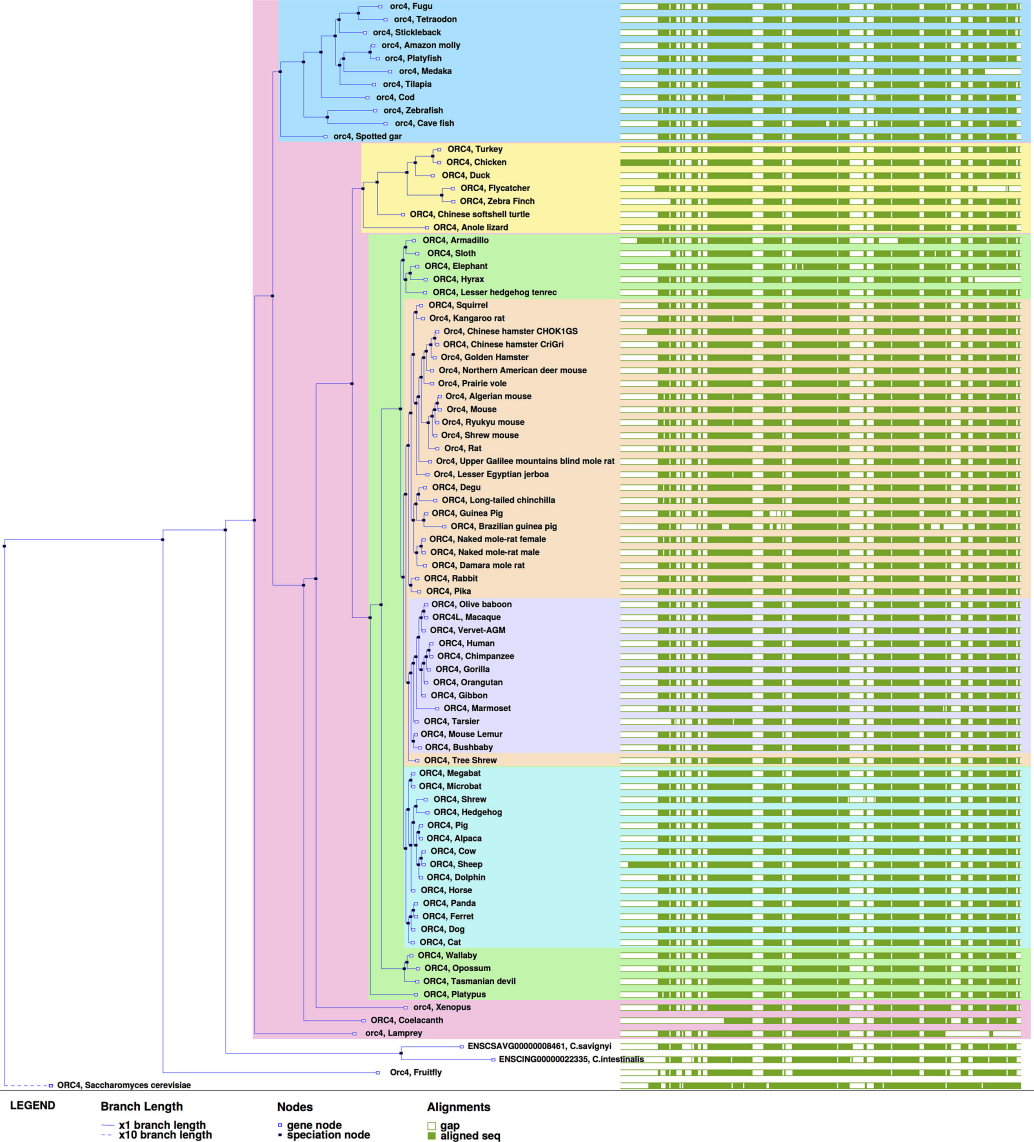
*ORC4* gene tree across 84 species.

Ensembl's comparative genomics resources have all been updated to include the new genomes and updated assemblies, as illustrated on Figure 1. This represents a 25% increase of the number of pairwise-alignments available, as all genomes are aligned to human, and all rodents to mouse. The multiple alignments have also been expanded, increasing the size of the Eutherian mammals EPO-LOW-COVERAGE multiple-alignment from 40 genomes to 55. We added both naked mole-rat genomes and both CHO genomes to the main set of trees, allowing direct orthology calls to mouse, human and other key species, and we added *Mus caroli* and *Mus pahari* to the Murinae gene-trees and orthologues set.

The addition of multiple genomes of the same species in the gene trees is still under development. In the initial implementation described here, we have decided not to unilaterally promote one assembly for a given species as representative of all the others available. Instead, we inserted all gene sequences into the trees independently although this leads to a simplified representation of evolution. Specifically, intra-species evolution is generally marked by recombination meaning that the history of the genes is correctly represented as a directed acyclic graph known as an Ancestral Recombination Graph (ARG) (24). As an intermediate step, we will soon update the gene trees in the relevant species so that relationship between the separate assemblies are appropriately labelled.

In our TreeFam gene homology resource, we increased the sensitivity of our orthology-calling methods, especially for short sequences. The proportion of proteins shorter than 50 amino-acids with at least one homologue rose from 43% to 50%, and the proportion of proteins shorter than 20 amino-acids with at least one homologue rose from 1.3% to 25%. All our protein-families and gene gain/loss trees can now be retrieved from our public REST API, which expands the available programmatic options beyond our Perl API.

We regularly update the links to external references for all 97 chordate species in Ensembl. For the newly added mouse strains, where little strain-specific external data is available, these links have been inferred from the reference mouse. Genes in a strain which had a one-to-one ortholog in the mouse GRCm38 reference assembly thus inherited links relevant to that gene. We also mapped murine microarray probes to all the different strain genomes.

## SUPPORT FOR GENOME INTERPRETATION

Our updates to the Ensembl REST server can be more frequent than the time taken to complete long-running research projects or applications that require consistent analysis against the same Ensembl release. For these reasons, we now maintain archives of the REST server starting with Ensembl release 87 (e.g. http://mar2017.rest.ensembl.org/). Archives will be available for at least five years from their initial release to enable consistent and reproducible analysis for publications and other genomic workflows.

**Figure 2.**
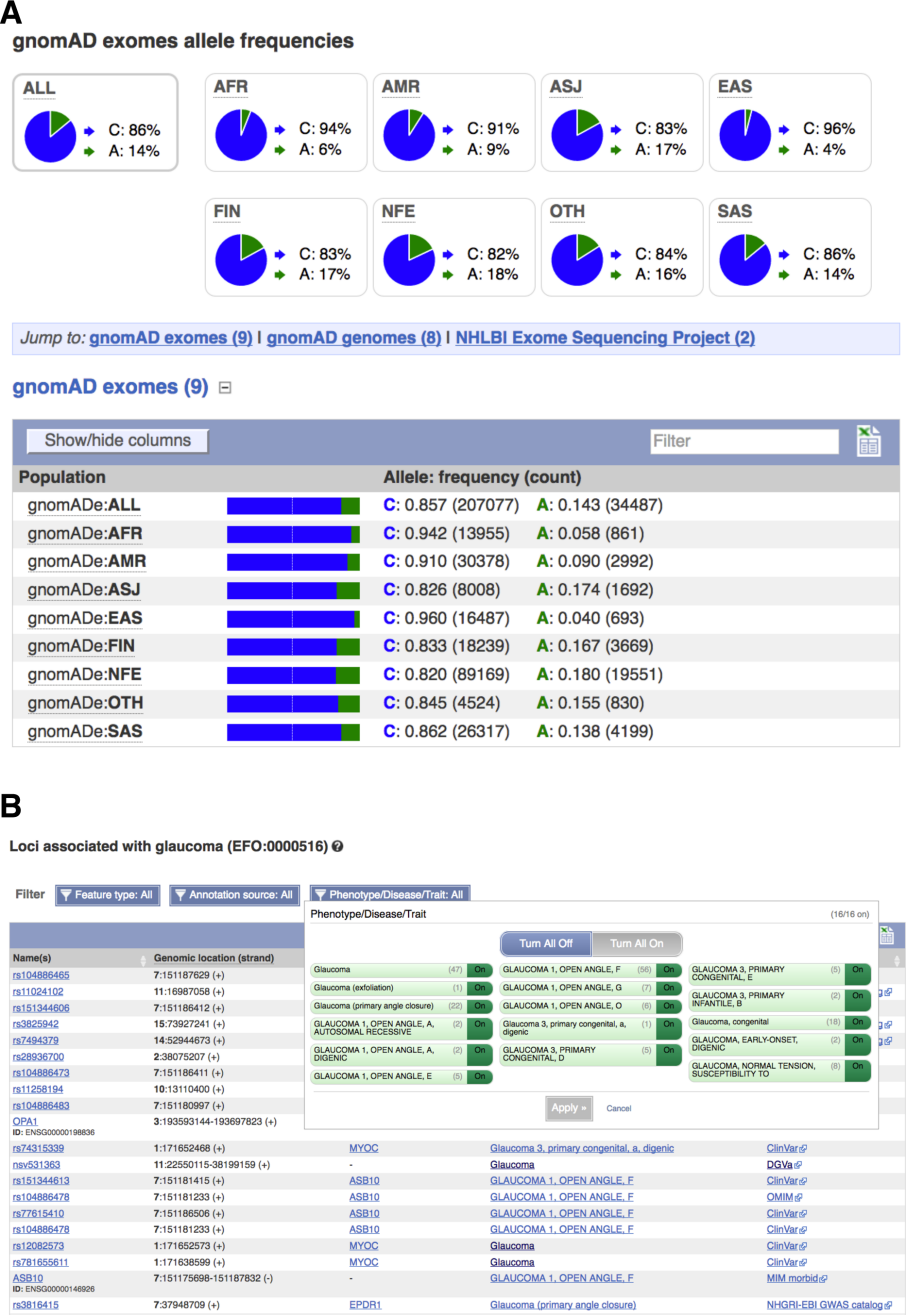
Detailed variant reports. (**A**) Variant minor allele frequencies of a given variant across gnomAD populations. (**B**) Variant association table, as returned by the ontology-aware search, that contains results closely related to the query, in this case ‘glaucoma’.

A number of large scale sequencing projects, such as the Genome Aggregation Database (gnomAD, http://gnomad.broadinstitute.org/), UK10K (25) and NHLBI Trans-Omics for Precision Medicine (TOPMed, https://www.nhlbi.nih.gov/research/resources/nhlbi-precision-medicine-initiative/topmed) projects, have made allele frequency data available this year in addition to the 1000 Genomes Phase 3 data (8). We have extended our API to efficiently produce frequency data from these cohorts. To help filter out common variants when performing association studies, we report the highest minor allele frequency observed in any population in the 1000 Genomes, gnomAD and TOPMed projects, both on our variant pages (see Figure 2A) and via our Perl API. We now also make linkage disequilibrium (LD) plots available for insertions and deletions on our website.

This year we significantly updated the VEP code to improve its robustness and functionality. In particular we enhanced our analysis of RefSeq human transcripts. Predicting the functional consequences of variants on RefSeq transcripts that differ from the reference assembly can be a challenge. To deliver more accurate results, the VEP now uses NCBI’s alignments of these transcripts onto the genome to expose any differences. Additionally, the VEP now predicts the impact of missense variants on the protein function of RefSeq transcripts using SIFT (26) and PolyPhen 2 (27). New plugins support more detailed descriptions of variants located near splice sites, loss of function intolerant scores for genes (28), and additional measures of variant deleteriousness (https://www.biorxiv.org/content/early/2017/06/12/148353).

We import phenotype associations from many different sources into Ensembl. Often, we encounter the same disease or trait in different databases under different labels (e.g. Type 2 diabetes and ‘diabetes, type II’). We now map these descriptions to ontology terms, thus bringing together records describing the same disease under different names, as well as different subtypes of traits and diseases sharing the same phenotypes. This improves the ability to query results aggregated across many sources. It also improves the legibility of our phenotype tables, which are now grouped by ontology term, as shown on Figure 2B. As these tables may contain hundreds of records, we added filters to display only selected results based on attributes such as locus type (e.g. genes or variants) or data source. For non-human species, in particular mouse and a number of livestock species, we now map to the Mammalian (https://github.com/obophenotype/mammalian-phenotype-ontology) and Clinical Measurement (29) ontologies respectively. New REST endpoints have been created to allow programmatic access to these mapped results across all species.

Similarly, variants can appear under a number of identifiers including dbSNP RefSNP identifiers, ClinVar (30) accessions and the Human Genome Variation Society (HGVS, 31) nomenclature at genomic, transcript or protein level. Identifiers used in past publications are often made obsolete over time, making it difficult to link them to current knowledge. To help address this problem, we have implemented a REST endpoint that returns all currently known identifiers for a given variant name. Many types of malformed and redundant HGVS descriptions are correctly interpreted and all possible variant identifiers returned.

In collaboration with RNAcentral and University College London, we added GO term annotations for some for non-coding transcripts as of Ensembl release 89 (May 2017). These supplement the protein-coding transcript GO terms Ensembl has included for many years from UniProt.

As the number of Ensembl's tools and services grows, we recognise the need for our infrastructure to be quickly and easily deployed locally. This supports use cases such as independent annotation (32) or clinical genetics applications that cannot send queries to our servers for privacy concerns. We now provide an automated deployment tool using Ansible (https://github.com/Ensembl/ensembl-rest-deploy) to go from a fresh VM to a ready-to-deploy Ensembl REST service. Similarly, the external dependencies of the Ensembl analysis methods can be installed very rapidly on any system using our Homebrew recipes (https://github.com/Ensembl/homebrew-ensembl). Both are already successfully used within the project to set up our analysis tools, deploy REST for a new release as well as for the VM available from the FTP site. These tools supplement parallel efforts such as GenomeHubs, which support rapid local deployment of the Ensembl databases and web server (33).

Finally, we now distribute intermediary results of our epigenomic processing pipeline, which are particularly useful for the high-throughput genome-wide reanalysis of non-coding variants. These include BigWig files of all the consistently mapped ChIP-seq datasets as well as segmentation BigBed files of all the epigenomes included in Ensembl.

## AN INTUITIVE BROWSING EXPERIENCE

**Figure 3.**
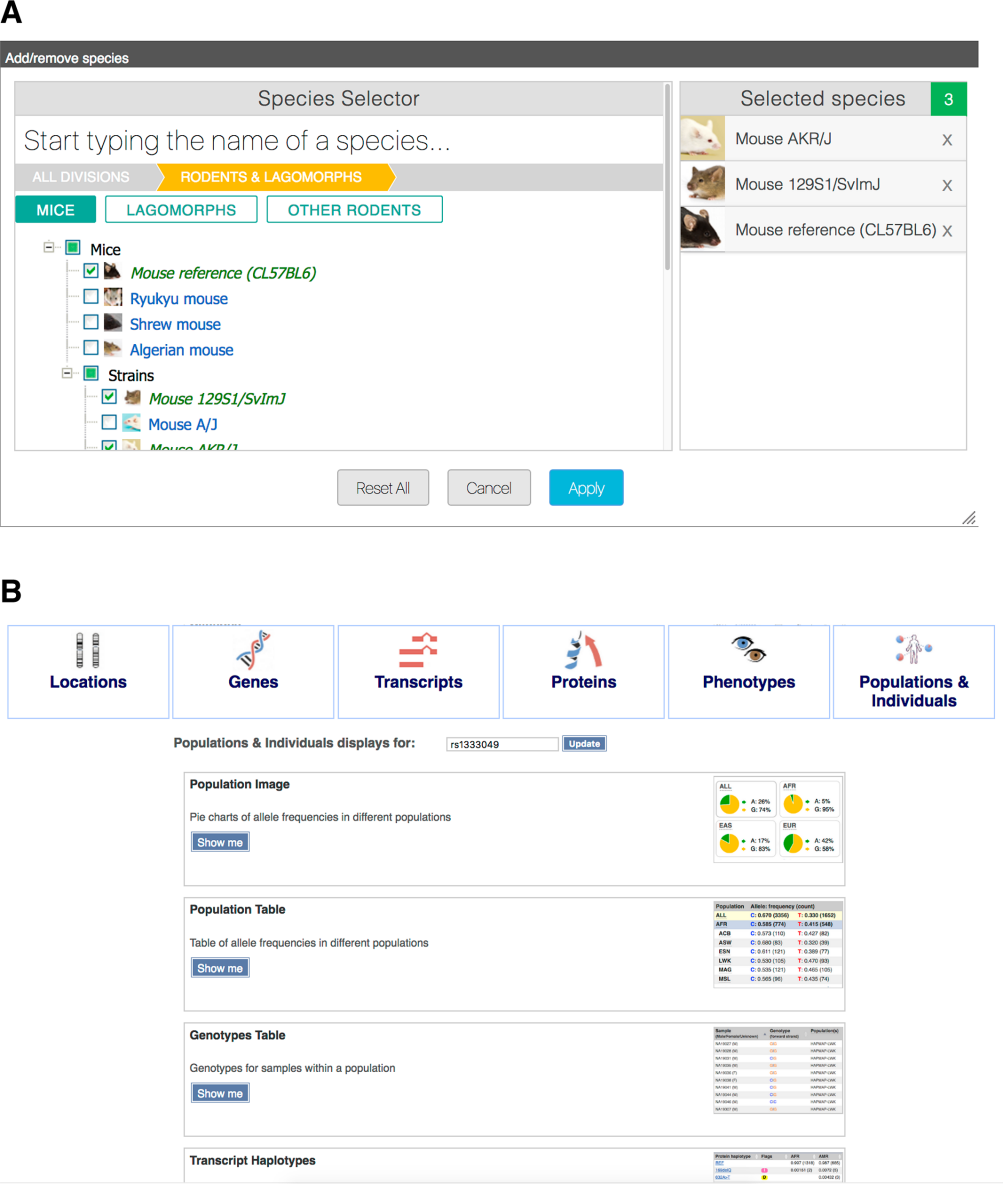
Quick selection menus. (**A**) The species selection tool can be used to quickly search for species by clade. (**B**) The plot gallery produces direct links to all Ensembl views and resources regarding a gene, variant or locus of interest.

A new gallery portal showcases the wealth of visual interfaces available in Ensembl (http://www.ensembl.org/info/website/gallery.html). Searches of the gallery return thumbnail images for all relevant views associated with specific genes, variants or genomic locations (see Figure 3B). Because of the number of views available, they are grouped by themes. For example, for a given SNP identifier, there are views relating to its region, overlapping transcripts, overlapping genes, overlapping protein sequences, associated phenotypes and population genetics. The gallery portal is designed to help newcomers discover unknown resources in Ensembl, as well as more experienced users jump directly to specific services.

A new interface to search for and select species within our tools and comparative views has been released. The new design (see Figure 3A) allows us to present the available species in a hierarchy of clades and we hope this will prove indispensable as the number of species increases over the next few years.

As we continue to deliver training courses around the world, we developed a new training website (http://training.ensembl.org) to distribute training materials from these courses, including slides, screenshot-by-screenshot walkthroughs of the website and hands-on exercises with answers. Materials from previous courses are available in perpetuity, allowing course participants to access them both during and after the course. The materials carry a Creative Commons BY license, allowing other trainers to use and adapt these materials for their own training. The site also includes information for hosts wanting to invite Ensembl for their own course, and links to courses that participants can register for.

## CONCLUSION

The Ensembl infrastructure is keeping up with the pace of cutting-edge genomics research, in scale, breadth and complexity. Thanks to robust engineering, we are developing new applications from our existing resources, and regularly upgrading the latter when needed. Our current priorities are scaling up to more species, delivering useful services for genome interpretation and improving the web interface. Given the unrelenting pace of genomics, we expect to be pursuing these efforts for years to come.

## AVAILABILITY

The Ensembl website (http://www.ensembl.org) provides access to all of our services and documentation, including the REST API (http://rest.ensembl.org) and BioMart (http://www.ensembl.org/biomart/). Ensembl imposes no restrictions on access to, or use of, the data provided and the software used to analyse and present it. All Ensembl code is available on Github (http://www.github.com/Ensembl/) under the Apache 2.0 licence.

Queries about hosting Ensembl workshops and any other questions about Ensembl can be directed to our helpdesk (helpdesk@ensembl.org). We can also be contacted informally via social media platforms, including Twitter (@ensembl) and Facebook (Ensembl.org). Our blog posts include detailed descriptions of every Ensembl release and other information (http://www.ensembl.info).
